# Novel Nutrition Profiling of New Zealanders’ Varied Eating Patterns

**DOI:** 10.3390/nu10010030

**Published:** 2017-12-31

**Authors:** Olivia Maclaren, Lisa Mackay, Grant Schofield, Caryn Zinn

**Affiliations:** 1 Eastern Institute of Technology, Napier 4112, New Zealand; 2 School of Sport and Recreation, Auckland University of Technology, Auckland 1010, New Zealand; lisa.mackay@aut.ac.nz (L.M.); grant.schofield@aut.ac.nz (G.S.); czinn@aut.ac.nz (C.Z.)

**Keywords:** nutrition, eating patterns, profiles, observational study, New Zealand

## Abstract

There is increasing recognition that the relationship between nutrition and health is influenced by complex eating behaviors. The aims of this study were to develop novel nutrition profiles of New Zealanders and to describe the prevalence of these profiles. Observational, cross-sectional data from the Sovereign Wellbeing Index, 2014 was used to develop the profiles in an a-priori process. Descriptive prevalence for the total data (*N* = 10,012; 4797 males; 18+ years) and profiles were reported. Nutrition question responses were presented as: Includers (consumed few time a week or more), Avoiders (few time a month) and Limiters (not eaten). Fruit or non-starchy vegetables were Included (fruit: 83.4%, 95% confidence interval (CI: 82.7, 84.1); vegetables: 82.6% (81.8, 83.4)) by the majority of the sample. Also Included were confectionary (48.6% 95% CI (47.6, 49.6)) and full sugar drinks (34.3% (33.4, 35.2)). The derived nutrition profiles were: Junk Food (22.4% 95% CI (21.6, 23.3)), Moderator (43.0% (42.1, 44.0)), High-Carbohydrate (23.0% (22.2, 23.8)), Mediterranean (11.1% (10.5, 11.8)), Flexitarian (8.8% (8.2, 9.4)), and Low-Carbohydrate (5.4% (4.9, 5.8)). This study suggests that New Zealanders follow a number of different healthful eating patterns. Future work should consider how these alternate eating patterns impact on public health.

## 1. Introduction

Nutrition, along with physical activity, is one of the major determinants of health and disease [1,2,3,4]. Yet there are a number of issues around the youthful science of public health nutrition that are still to be addressed. One of these is the increasing recognition that the relationship between nutrition and health is influenced by complex eating behaviors and patterns [5]. The more traditional focus on individual nutrient intake is limited in its ability to assess multiple potential interactions [6,7]. A number of authors have argued the benefits of examining dietary patterns as they more closely resembles “real-word” behaviors [5,7,8]. It has also been suggested that a more integrated approach that includes various social science viewpoints is an important future direction for understanding the complexities of nutritional science [9]. This study utilizes a social science viewpoint to broadly describe eating behaviors as a novel approach to the epidemiological study of nutrition and public health.

The impact of overall dietary patterns rather than isolated nutrient intake has increasingly been shown to have importance to metabolic health [5]. Some studies have examined patterns similar to dietary guidelines and the relationship to heart disease. In one study a “Prudent” dietary pattern was linked to a lower risk of coronary heart disease compared to a “Western” dietary pattern [10,11]. In another study the United Kingdoms’ dietary guidelines showed a reduction in risk factors for cardiovascular disease compared to more traditional British eating patterns [12].

Two alternate eating patterns that have also increasingly been examined are the Mediterranean and DASH (Dietary Approaches to Stop Hypertension) diets [5]. A number of meta-analyses have linked the Mediterranean dietary pattern to a reduced risk of coronary heart disease, myocardial infarctions, stroke [13], hypertension [14], metabolic syndrome [15], and diabetes [16,17]. The DASH diet has also been linked to reduced risk of diabetes [17] hypertension [14] and cardiometabolic risk factors [18]. However, some concerns have been raised about the quality of the evidence [19,20].

Other alternate patterns have yet to be studied, however, when food or nutrient-focused studies are examined there are indications that alternate patterns may have benefits to health. For example, carbohydrate restriction has shown evidence of weight loss [21], reduced risk of cardiovascular disease and total mortality [22], along with reductions in diabetic symptoms [23]. Reductions in high-sugar foods and drinks have also been associated with reductions in body weight [24], and have therefore, been linked to reduced risk of non-communicable diseases [25]. Vegetarianism and the permutation of various meat restrictions appear to have equivocal benefits to health, likely due to the large variations in food quality that can be incorporated under the meat restrictive banner [5,26,27]. The next step, therefore, is to develop dietary patterns that incorporate more alternate approaches outside of governmental guidelines, but include food groups linked to good health as described above.

In New Zealand, the governmental guidelines on healthy eating [28], like most developed countries, apply a food-specific approach to what is prescribed and what should be avoided. The guidelines emphasize a diet consisting of predominantly carbohydrates such as fruit, vegetables, and wholegrains; some protein such as lean meats, nuts and seeds, and low-fat or reduced-fat dairy products; and limiting saturated fats of predominantly animal origin. Additionally, they suggest limiting the intake of added salt and sugars [28]. Because of this narrow focus on what constitutes a healthy diet, the monitoring of population nutrition in New Zealand to date has also been limited to whether these recommendations are or are not being followed [29,30,31,32]. With the wealth of knowledge available on the internet, individuals are undoubtedly being exposed to alternate eating paradigms. A recent analysis of popular online books and podcasts reported the most popular nutrition philosophies were low-carbohydrate and vegetarian approaches [33]. This demonstrates an interest in alternate eating patterns, but what we currently do not know is how many people put this interest into practice.

This study, therefore, incorporated two key aims: (i) to use a simple survey, incorporated as part of the Sovereign Wellbeing Index (SWI) [34], to develop novel nutrition profiles of New Zealanders that reflect a broad range of eating patterns; and (ii) to describe the prevalence of these nutrition profiles to provide a broader behavioral viewpoint of New Zealanders eating patterns.

## 2. Materials and Methods

### 2.1. Participants

Observational cross-sectional data from the SWI, Round 2 (2014) [34] was used in this study. Participants were recruited through the largest commercial database in New Zealand which ensured complete anonymity for respondents. Round 2 of the SWI comprised 10,012 participants (15.7% response rate). The representativeness of the sample is discussed below. All participants gave their informed consent for inclusion before they participated in the study. The study was conducted in accordance with the Declaration of Helsinki, and the protocol was approved by the Auckland University of Technology Ethics Committee (12/201).

### 2.2. Data Collection

Participants completed the entire SWI web-based survey on wellbeing (65 items), health and lifestyle (64 items), and demographics (20 items), which took around (median) 21 min to complete. Data was collected in the middle of the New Zealand spring season between 1 October 2014 and 3 November 2014 (33 days). The demographic (age, gender, labor force status, ethnicity, average household income) and nutrition food profiling questions, as described below, was used to develop the nutritional profiles. The remainder of the data from the SWI is described elsewhere [34].

There were 21 food profiling items which examined the consumption patterns of major food groups to determine whether different food groups were restricted or included in the participants’ diets. These questions addressed participants’ food consumption using the following leader; “On average, over the last four weeks, how often have you consumed the following food?” Six response options were available to participants (Table 1). Responses were classified into three consumption patterns (Avoiders, Limiters, Includers) for each food profiling question for statistical analysis of prevalence. Avoiders were defined as having not eaten a food group, Limiters were defined as consuming a food group a few times a month, and Includers were defined by consumption a few times a week or more often. The nutrition survey questions have previously been content-validated and reliability-tested [35]. Quadratic weighted kappa for test-retest reliability showed fair to excellent strength of agreement for 20 out of the 21 nutrition survey questions.

### 2.3. Profiling Procedures

Novel nutrition profiles were devised through an investigator-driven process utilizing an expert panel. The panel represented a wide range of expert knowledge in the areas of public health, nutrition, and physical activity and included a New Zealand Registered Dietitian, a public health academic specializing in physical activity and nutrition, an exercise physiologist, and two epidemiologists. Investigator-driven profiling methodology was chosen in preference to data-driven clustering analysis, as the aim of this study was to develop and report on the prevalence rates of nutrition patterns linked to positive health outcomes and those common in the popular media.

Initially, some time was spent developing a short list of possible nutrition profiles and selecting the relevant question from the survey to differentiate these profiles. Six profiles were selected, based on current popular eating approaches and governmental dietary guidelines. The profile groups developed were:

Junk Food Group: This group was classified based on the daily consumption of “junk” type foods such as takeaway food, confectionery, and sugary drinks. All the other nutrition profiles were developed from the remainder of the sample once the Junk Food Group and therefore, the high inclusion of “junk” type foods, had been removed.

Flexitarian Group: This group was based on the irregular or non-consumption of white, red and processed meat and was designed to include as many meat restricting groups as possible, such as; ovo-vegetarians, vegetarians, and vegans, and both strict and flexible followers.

High-Carbohydrate Group: This group was classified based on the regular consumption of non-starchy vegetables and grains.

Mediterranean Group: This was a subset of the High-Carbohydrate group and was based on the traits of a Mediterranean diet, which included regular consumption of non-starchy vegetables, grains, olive oil, and either white meat or fish [36].

Low-Carbohydrate Group: This group was classified based on the regular consumption of non-starchy vegetables and a limited consumption of grains.

Moderator Group: The remainder of the sample was classified as the Moderator group, which consumed most of the different food types.

**Figure 1:**
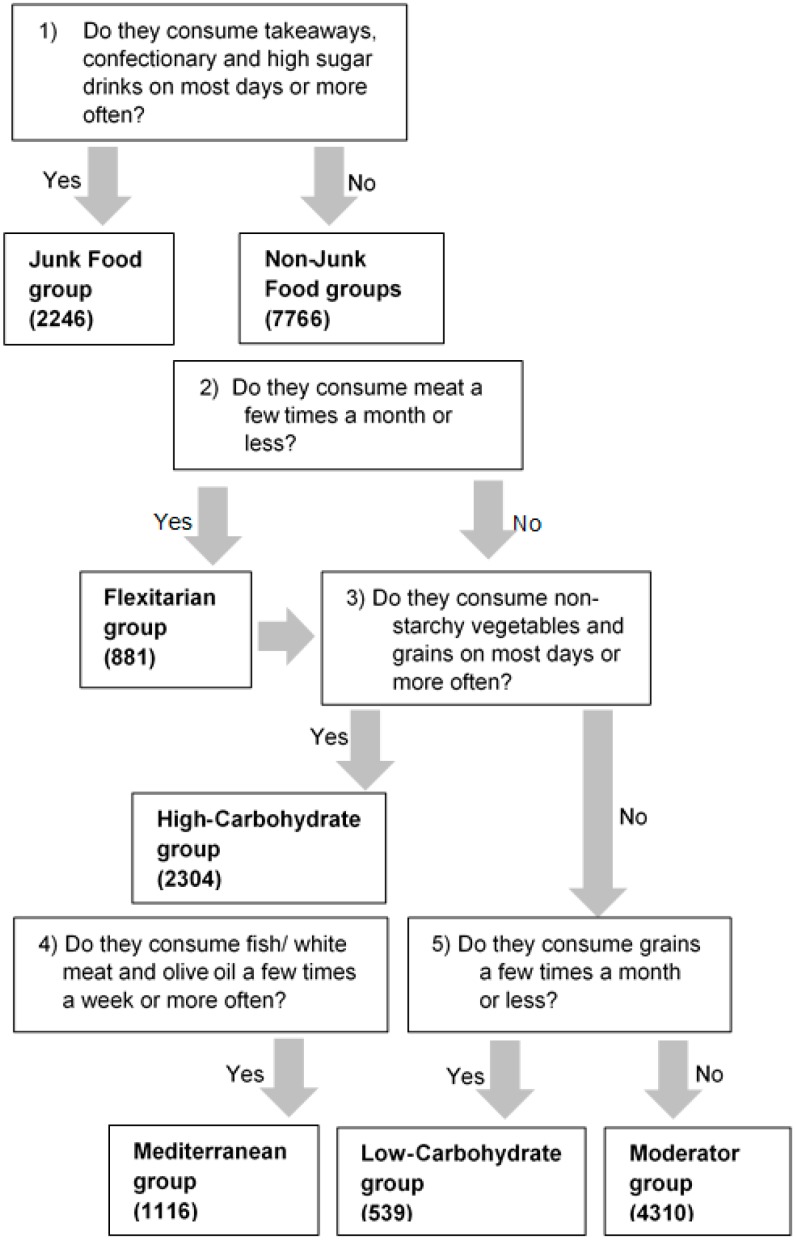
Questions from the Sovereign Wellbeing Index (2014) used to develop six novel nutrition profiles ^1^. ^1^ Numbers in brackets are the nutrition profile group size (*n*).

The questions selected from the nutrition section of the SWI that were used to differentiate the profiles in a stepwise approach are shown in Figure 1. Due to this approach, some participants could be classified into more than one group. For example, it was possible for participants profiled into the Flexitarian group to also be profiled into either the High-Carbohydrate, Mediterranean or Low-Carbohydrate groups. Additionally, the Mediterranean group was a sub-group of the High-Carbohydrate group. Full details on the nutrition questions and response options are shown in Table 1.

### 2.4. Data Analysis

Descriptive statistics were used to describe both the profile groups and responses to the individual nutrition questions. Incomplete or non-response data were excluded on a per question basis. This included system missing data and responses of “prefer not to answer”. Further details on data handling for the full survey can be found in [37].

Survey data were analyzed using IBM SPSS Statistics (version 24, Armonk, NY, USA). The SPSS custom tables function was used to describe the total sample simple prevalence (frequency counts and percentage) for each nutrition question. The SPSS syntax editor was used to profile the data into the six nutrition profiles (Junk Food, Low Carbohydrate, High Carbohydrate, Flexitarian, Mediterranean, Moderator groups) from specific question responses as shown in Figure 1.

The SPSS crosstabs function was used to derive the descriptive prevalence estimates (frequency counts and percentage) for the nutrition profiles. Cross-tabulations were also used to determine the overlap between profile groups. A margin of error around the prevalence estimates was indicated using 95% confidence intervals (CI). Results are given as % (95% CI) unless otherwise stated.

## 3. Results

### 3.1. Demographics of Total Sample

The demographics of the participants from Round 2 of the SWI which were used to develop the nutrition profiles showed a predominant European ethnicity, and were predominantly in employment. The gender, household income, and age distribution of participants were fairly uniform, except for a smaller sample group in the under 20-year age group, and a larger group in the under $30,000 income bracket (Table 2). When the SWI data was compared to the New Zealand 2013 census probability samples [38], similar prevalence were seen for gender (% variance; males 0.6, females −0.6), age (% variance range; −2.0 to 1.7), ethnicity (% variance range; −0.8 to 5.8), and labor force status (% variance range; −3.9 to 0.2). Smoking status was also similar (% variance; smokers 0.5, non-smokers −0.5).

### 3.2. Prevalence of the Nutrition Profile Groups

**Figure 2:**
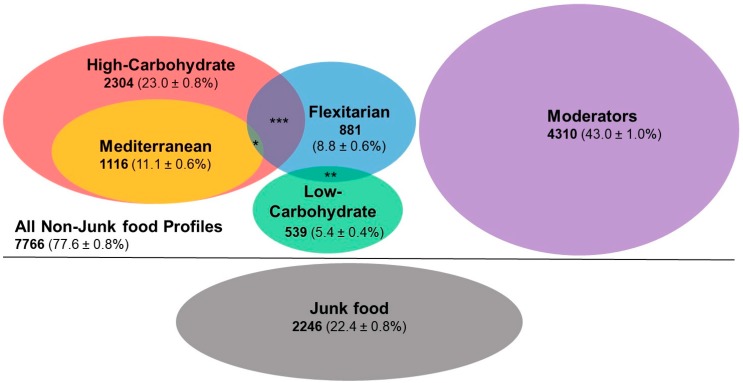
Nutrition profiles derived from the Sovereign Wellbeing Index, 2014. Total sample *N* = 10,012; totals are given for each profile group with % ± 95% confidence limits in brackets; Crossovers percent are percentage of total sample: * High Carb, Mediterranean & Flexitarian 23 (0.2 ± 0.1%); ** Flexitarian & Low Carb 52 (0.5 ± 0.1%); *** High Carb & Flexitarian 216 (2.2 ± 0.3%).

Figure 2 presents the nutrition profiles, indicating overlaps where appropriate. The Junk Food Group contained almost a quarter of the sample; of the other five profile groups, the largest was the Moderator group, with the Low-Carbohydrate group being the smallest. There was some overlap between profile groups, with the largest overlap occurring between the Flexitarian and High-Carbohydrate groups.

### 3.3. Demographics of the Nutrition Profile Groups

A greater percentage of females were in the High-Carbohydrate (55.5%, 95% CI (53.4, 57.5)), Low-Carbohydrate (61.3%, (57.1, 65.4)), Mediterranean (57.6% (54.7, 60.5)), and Flexitarian (56.2% (52.9, 59.5)) groups, compared to the total sample (51.0%). The Junk Food Group showed similar prevalence across genders (females: 50.0% (47.9, 52.1)), and the Moderator group showed a slightly greater number of males (51.2% (49.7, 52.7)). There were also some differences in age group distributions across food profiles. The 20–29 years age group was over-represented in the Junk Food Group (26.8% (24.9, 28.8)), whereas the 50–59 years and 60+ years age groups were over-represented in the Low-Carbohydrate group (27.9% (23.8, 31.9), 25.1% (21.2, 29.0) respectively). The 60 years and over was over-represented in the High-Carbohydrate group (23.4% (21.5, 25.2)).

### 3.4. Prevalence for Individual Nutrition Questions

Table 3 presents the prevalence of Avoiders, Limiters, and Includers for each food profiling question across the profile groups. Across the total sample, 16.6% Avoided or Limited fruit, and 17.4% Avoided or Limited non-starchy vegetables. A high proportion of the sample Included confectionery and full-sugar drinks in their diets on a regular basis. Of the animal proteins, fish and shellfish were the most commonly Avoided or Limited. Grains were Included regularly in the diet for the majority of the sample.

The Flexitarian profile group had a higher prevalence of grain Limiters than the total sample. The Flexitarian group also restricted a number of different food groups in addition to animal product food groups.

The Moderator profile group had a higher prevalence of grain Includers compared to the total sample. The Moderator group had a pattern of a high prevalence of Includers across many of the food groups.

Though grain restriction was a profiling question for the Low-Carbohydrate group there were more Avoiders than Limiters for this food group. Additionally, the prevalence of Includers for starchy vegetables was lower than for the total sample. Though frequent consumption of confectionery and full sugar drinks were excluded from all groups except the Junk Food Group during the profiling process, the Low-Carbohydrate group showed the highest prevalence of Avoiders for these two food groups across all the other nutrition profiles.

In addition to the classification questions for the Junk Food profile, this group had the highest prevalence of Includers for butter and non-butter spreads, processed meat, and cakes and biscuits. Like the Moderator group, the Junk Food Group had a pattern of a high prevalence of Includers across a number of food groups.

The High-Carbohydrate group had a similar pattern to its sub-group, the Mediterranean profile group. The greatest prevalence of takeaway Limiters was in the High-Carbohydrate group. The Mediterranean group had the highest prevalence of olive oil Includers which was a classification question for this group. This group had the second highest prevalence of confectionery Includers.

## 4. Discussion

This study proposed a novel profiling system to examine New Zealander eating behaviors. A key finding was that the majority of New Zealanders include some form of “healthful” behavior most of the time. Three-quarters of the sample included food or food groups regularly that previous research has linked to improved metabolic health. However, a quarter of the sample was classified into the Junk Food Group and was therefore considered to have an “unhealthful” behavior pattern.

The profiling of nutrition behaviors that include patterns outside of the current governmental guidelines has been called for by a number of authors [5,7,8]. Ten years ago [9], a call was made for a shift away from nutrients to a more food-focused science of nutrition. This is beginning to occur and recent work has begun to show that cardiometabolic diseases are influenced by foods and combinations of foods in overall dietary patterns, rather than by individual nutrients [5]. Additionally, a multidisciplinary approach to nutrition that includes a social science paradigm has been suggested as a move towards understanding the complex interactions between eating behavior and the health consequences of those behaviors [9].

This study is the first that the authors are aware of that has attempted to describe a broad range of eating behavior patterns and included alternate patterns such as low-carbohydrate eating. This approach offers a way forward to help gain further insight into population health and eating and as a potential avenue towards future health promotion.

### 4.1. Future Directions

This study is an initial step in the observation of alternate eating paradigms in New Zealanders. Further work is still required to help understand the motivations behind various food choices to understand whether individuals are consciously choosing to follow specific dietary patterns and if so why. Genetic variations undoubtedly play a role in food choice, as well as the impact on the resulting health outcomes. The research field of epigenetics, nutrigenetics, and nutrigenomics [39] are likely to provide some interesting future implications around individualised food choices and may help us understand why certain eating patterns work better for some individuals than others. Future approaches to public health research should consider incorporating a broader approach in order to move towards a more positive health paradigm. More work in this area is now required. Although this work is specific to New Zealand, future comparisons should be made with other developed countries with similar governmental nutrition guidelines.

### 4.2. Study Limitations

Several study limitations should be noted. This is an observational study and therefore can identify trends that would benefit from further study; however, causal relationships cannot be inferred. Also, like all self-reported nutrition data, under-reporting of foods should be a consideration [40]. Seasonality may also have impacted on the results, as the data was collected over the New Zealand spring season.

The brevity of the survey questions was both a benefit, in that it increased the potential sample size by reducing cognitive load as well as increasing ease of collection; however, it also limited the detail that could be delineated from the data. If the definition of groups had been made more specific, the size of some of the profile groups would have been very small. Therefore, groups such as vegetarian and vegans were included in a single profile (Flexitarian), and this may have led to the overall group pattern of exclusion. This may be also an explanation for some of the other unexpected patterns of exclusion or inclusion seen across profile groups.

Due to the step-wise profiling process, the size of all the profile groups apart from the Junk Food Group and the default Moderator group may actually be larger than described here. The Junk Food profile was defined first and excluded any participants that consumed takeaways, full sugar drinks, and confectionary daily from the other profile groups, even if they followed any other dietary patterns. Additionally, the definitions of the dietary patterns profiled in this study were consciously broad and based on the fundamental characteristics of the various eating patterns. Currently, dietary patterns are not well defined and, therefore, the wider definitions used here may have described larger groups than those that consciously follow specific eating patterns.

The nutrition questions included in the SWI were reviewed for re-test reliability and content validity [35]; however, due to the timing of the SWI, modification of questions was not possible prior to data collection. The nutrition profiling question on full sugar drinks showed poor agreement for test-retest reliability. This question was used as a key profiling question for separation of the Junk Food Group from the remainder of the nutrition profiles. This is acknowledged as limitation could affect the size of the profile groups.

Finally, this study involved an investigator-driven approach to profiling as selected dietary patterns were the focus of this study. Though not necessarily a limitation, this requires acknowledgement and a suggestion that a future line of inquiry may be an interview-based validation of the profiling process used here.

## 5. Conclusions

Since the current population level monitoring surveys in New Zealand [30,31,32,41] are predominantly designed around understanding to what extent the governmental eating guidelines have been met, nutritional information is typically gathered via an interviewer-driven dietary history, comprising of a 24 h diet recall and a food frequency questionnaire. Foods are then quantified as healthy or unhealthy as defined by the guidelines [28]. The data itself provides a good account of individual food intake, but this provides only a narrow view of nutrition patterns or approaches. This study indicates that New Zealanders follow a number of different eating patterns, that could be considered healthful; therefore, a more comprehensive approach to monitoring is needed in order to more fully understand how these alternate eating patterns impact on public health.

## Figures and Tables

**Table 1:** Food profiling questions and response options from the Sovereign Wellbeing Index, 2014.

Questions	Response Options
*On average over the past 4 weeks, how often have you consumed the following food?* ▪ All grain products (including rice, pasta, cereals, any type of grain based bread) ▪ Full fat dairy products (including cheese, milk, and yoghurt) ▪ Butter ▪ Low-fat dairy products (including cheese, milk, and yoghurt) ▪ Eggs ▪ Margarine or other non-butter spreads (including Olivani, Flora Pro Active) ▪ Oils: olive, avocado, macadamia, or coconut ▪ Oils: any other vegetable oil (including sunflower, rice-bran oil, canola, peanut, soy) ▪ Red meat (including beef, lamb, venison) ▪ White meat (including chicken, pork, turkey) ▪ Protein powders and/or bars ▪ Processed meat (including salami, sausages) ▪ Fish and shellfish ▪ Fruit ▪ Starchy vegetables (including potatoes, kumara, yams) ▪ All other non-starchy vegetables ▪ Cakes, biscuits, chips, crackers, or muesli ▪ Nuts ▪ Confectionary (including sweets and chocolate) ▪ Full sugar soft drinks, sports drinks, fruit juice or cordial ▪ Takeaways (including fast food outlets, fish and chips)	▪ I haven’t eaten it [A] ▪ A few times a month (1–3 times a month) [L] ▪ A few times a week (1–3 times a week) [I] ▪ On most days [I] ▪ At most meals [I] ▪ Prefer not to answer

A = Avoider, L = Limiter, I = Includer.

**Table 2:** Demographic data of respondents ^1^ to the Sovereign Wellbeing Index, 2014.

	*N*	%
Total Population	10,012	100.0
**Gender**	9904	98.9
Male	4797	47.9
Female	5107	51.0
**Age**	8614	86.0
under 20 years	270	2.7
20–29 years	1692	16.9
30–39 years	1602	16.0
40–49 years	1655	16.5
50–59 years	1694	16.9
60 years and over	1701	17.0
**Ethnicity ^1^**	10,444	97.4
Maori	956	8.9
European	7605	70.9
Pacific people	310	2.9
Asian	1269	11.8
Other	304	2.8
**Labour Force Status**	9613	96.0
Employed	5503	55.0
Unemployed	714	7.1
Not in the labour force ^2^	2822	28.2
Other	574	5.7
**Quintiles of Household Income**	7654	76.4
≤$30,000	1821	18.2
$30,001–$50,000	1456	14.5
$50,001–$70,000	1305	13.0
$70,001–$100,000	1535	15.3
≥$100,001	1537	15.4

^1^ Participants could select more than one ethnicity; ^2^ Neither employed nor unemployed (including retired people, students, home duties, or physical or mental impairment).

**Table 3:** The prevalence of different food groups across profile groups ^1^.

Food Group	Consumpt-Ion Level ^3^	Total Sample			Junk			Flexitarian			Mediterranean			Low-Carbohydrate			High-Carbohydrate			Moderator		
		*n*	%	95% CI ^2^	*n*	%	95% CI	*n*	%	95% CI	*n*	%	95% CI	*n*	%	95% CI	*n*	%	95% CI	*n*	%	95% CI
	*Total N*	*10,012*			*2246*			*881*			*1116*			*539*			*2304*			*4310*		
All grain products	Avoiders	413	4.3	(3.9–4.7)	102	4.6	(3.8–5.5)	57	6.5	(5.0–8.3)	0	0.0	(0.0–0.0)	116	21.5	(18.2–25.1)	0	0.0	(0.0–0.0)	145	3.7	(3.1–4.3)
	Limiters	1371	14.3	(13.6–15.0)	290	13.0	(11.7–14.5)	208	23.7	(21.0–26.6)	0	0.0	(0.0–0.0)	423	78.5	(74.9–81.8)	0	0.0	(0.0–0.0)	495	12.6	(11.6–13.7)
	Includers	7824	81.4	(80.6–82.2)	1837	82.4	(80.8–84.0)	612	69.8	(66.7–72.8)	1116	100.0	(100.0–100.0)	0	0.0	(0.0–0.0)	2304	100.0	(100.0–100.0)	3287	83.7	(82.5–84.8)
Full-fat dairy products	Avoiders	951	9.9	(9.3–10.5)	137	6.1	(5.2–7.2)	154	17.5	(15.1–20.1)	112	10.0	(8.4–11.9)	89	16.5	(13.6–19.8)	281	12.2	(10.9–13.6)	354	9.0	(8.2–9.9)
	Limiters	1531	15.9	(15.2–16.6)	281	12.6	(11.3–14.0)	246	28.0	(25.1–31.0)	137	12.3	(10.5–14.3)	112	20.8	(17.5–24.4)	294	12.8	(11.5–14.2)	649	16.5	(15.4–17.7)
	Includers	7125	74.2	(73.3–75.1)	1814	81.3	(79.6–82.8)	479	54.5	(51.2–57.8)	866	77.7	(75.2–80.0)	338	62.7	(58.6–66.7)	1726	75.0	(73.2–76.7)	2921	74.4	(73.1–75.8)
Butter	Avoiders	2612	27.2	(26.3–28.1)	523	23.5	(21.7–25.3)	322	36.6	(33.5–39.8)	226	20.3	(18.0–22.7)	169	31.4	(27.5–35.4)	611	26.5	(24.8–28.4)	1078	27.5	(26.1–28.9)
	Limiters	2461	25.6	(24.7–26.5)	470	21.1	(19.4–22.8)	284	32.3	(29.2–35.4)	304	27.2	(24.7–29.9)	128	23.7	(20.3–27.5)	640	27.8	(26.0–29.7)	1024	26.1	(24.8–27.5)
	Includers	4530	47.2	(46.2–48.2)	1235	55.4	(53.4–57.5)	274	31.1	(28.1–34.3)	586	52.5	(49.6–55.4)	242	44.9	(40.7–49.1)	1051	45.7	(43.6–47.7)	1820	46.4	(44.8–48.0)
Low-fat dairy products	Avoiders	2216	23.1	(22.3–23.9)	471	21.2	(19.6–23.0)	269	30.5	(27.6–33.6)	213	19.1	(16.9–21.5)	181	33.6	(29.7–37.6)	502	21.8	(20.2–23.5)	872	22.2	(21.0–23.6)
	Limiters	1723	18.0	(17.2–18.8)	383	17.3	(15.7–18.9)	229	26.0	(23.2–29.0)	135	12.1	(10.3–14.1)	99	18.4	(15.3–21.8)	298	13.0	(11.6–14.4)	757	19.3	(18.1–20.6)
	Includers	5654	58.9	(57.9–59.9)	1366	61.5	(59.5–63.5)	383	43.5	(40.2–46.8)	767	68.8	(66.0–71.5)	259	48.1	(43.9–52.3)	1501	65.2	(63.3–67.2)	2291	58.4	(56.9–60.0)
Eggs	Avoiders	623	6.5	(6.0–7.0)	171	7.7	(6.6–8.8)	151	17.2	(14.8–19.8)	25	2.2	(1.5–3.2)	42	7.8	(5.8–10.3)	120	5.2	(4.4–6.2)	193	4.9	(4.3–5.6)
	Limiters	2502	26.0	(25.1–26.9)	534	24.0	(22.2–25.8)	358	40.7	(37.5–44.0)	203	18.2	(16.0–20.5)	131	24.3	(20.9–28.1)	528	22.9	(21.3–24.7)	1035	26.4	(25.0–27.8)
	Includers	6481	67.5	(66.6–68.4)	1523	68.4	(66.4–70.3)	370	42.1	(38.9–45.4)	888	79.6	(77.1–81.9)	365	67.8	(63.8–71.7)	1654	71.9	(70.0–73.7)	2698	68.7	(67.3–70.2)
Margarine or other non-butter spreads	Avoiders	2470	25.7	(24.8–26.6)	478	21.5	(19.9–23.3)	303	34.5	(31.4–37.7)	307	27.5	(24.9–30.2)	223	41.4	(37.3–45.6)	644	28.0	(26.2–29.8)	930	23.7	(22.4–25.0)
	Limiters	1357	14.1	(13.4–14.8)	272	12.2	(10.9–13.7)	215	24.5	(21.7–27.4)	115	10.3	(8.6–12.2)	76	14.1	(11.4–17.2)	267	11.6	(10.3–13.0)	577	14.7	(13.6–15.8)
	Includers	5774	60.1	(59.1–61.1)	1471	66.2	(64.2–68.2)	361	41.1	(37.9–44.3)	694	62.2	(59.3–65.0)	240	44.5	(40.4–48.7)	1391	60.4	(58.4–62.4)	2421	61.6	(60.1–63.1)
Oils: olive, avocado, macadamia, coconut	Avoiders	2796	29.2	(28.3–30.1)	697	31.4	(29.5–33.4)	310	35.2	(32.1–38.4)	0	0.0	(0.0–0.0)	147	27.4	(23.7–31.3)	524	22.8	(21.1–24.6)	1191	30.4	(29.0–31.8)
	Limiters	2283	23.8	(22.9–24.7)	462	20.8	(19.2–22.5)	267	30.3	(27.3–33.4)	0	0.0	(0.0–0.0)	108	20.1	(16.9–23.7)	522	22.7	(21.1–24.5)	981	25.0	(23.7–26.4)
	Includers	4508	47.0	(46.0–48.0)	1061	47.8	(45.7–49.9)	304	34.5	(31.4–37.7)	1116	100.0	(100.0–100.0)	282	52.5	(48.3–56.7)	1250	54.4	(52.4–56.5)	1749	44.6	(43.1–46.2)
Oils: any other vegetable oil	Avoiders	1902	19.8	(19.0–20.6)	388	17.4	(15.9–19.1)	229	26.1	(23.2–29.0)	198	17.8	(15.6–20.1)	167	31.0	(27.2–35.0)	389	16.9	(15.4–18.5)	783	20.0	(18.8–21.3)
	Limiters	2316	24.2	(23.3–25.1)	476	21.4	(19.7–23.1)	312	35.5	(32.4–38.7)	171	15.3	(13.3–17.5)	117	21.7	(18.4–25.4)	472	20.5	(18.9–22.2)	994	25.4	(24.0–26.7)
	Includers	5372	56.0	(55.0–57.0)	1360	61.2	(59.1–63.2)	338	38.5	(35.3–41.7)	746	66.9	(64.1–69.6)	254	47.2	(43.0–51.4)	1437	62.5	(60.5–64.5)	2142	54.7	(53.1–56.2)
Red meat	Avoiders	674	7.0	(6.5–7.5)	140	6.3	(5.3–7.4)	356	40.4	(37.2–43.7)	38	3.4	(2.5–4.6)	35	6.5	(4.6–8.8)	193	8.4	(7.3–9.6)	109	2.8	(2.3–3.3)
	Limiters	1479	15.4	(14.7–16.1)	270	12.1	(10.8–13.5)	525	59.6	(56.3–62.8)	92	8.2	(6.7–10.0)	83	15.4	(12.5–18.6)	264	11.5	(10.2–12.8)	446	11.4	(10.4–12.4)
	Includers	7454	77.6	(76.8–78.4)	1816	81.6	(79.9–83.2)	0	0.0	(0.0–0.0)	986	88.4	(86.4–90.1)	421	78.1	(74.5–81.4)	1845	80.1	(78.5–81.7)	3372	85.9	(84.8–86.9)
White meat	Avoiders	478	5.0	(4.6–5.4)	92	4.1	(3.4–5.0)	303	34.4	(31.3–37.6)	13	1.2	(0.7–1.9)	31	5.8	(4.0–8.0)	132	5.7	(4.8–6.7)	52	1.3	(1.0–1.7)
	Limiters	1252	13.0	(12.3–13.7)	235	10.5	(9.3–11.9)	578	65.6	(62.4–68.7)	26	2.3	(1.6–3.3)	55	10.2	(7.9–13.0)	210	9.1	(8.0–10.3)	310	7.9	(7.1–8.8)
	Includers	7883	82.0	(81.2–82.8)	1902	85.3	(83.8–86.8)	0	0.0	(0.0–0.0)	1077	96.5	(95.3–97.5)	453	84.0	(80.8–87.0)	1961	85.1	(83.7–86.6)	3567	90.8	(89.9–91.7)
Protein powders and or bars	Avoiders	7049	73.5	(72.6–74.4)	1475	66.5	(64.5–68.5)	712	81.0	(78.3–83.5)	826	74.1	(71.5–76.7)	416	77.6	(73.9–81.0)	1815	78.9	(77.2–80.6)	2856	72.9	(71.4–74.2)
	Limiters	1138	11.9	(11.3–12.5)	295	13.3	(11.9–14.8)	102	11.6	(9.6–13.8)	116	10.4	(8.7–12.3)	47	8.8	(6.6–11.4)	222	9.7	(8.5–10.9)	493	12.6	(11.6–13.6)
	Includers	1397	14.6	(13.9–15.3)	447	20.2	(18.5–21.9)	65	7.4	(5.8–9.3)	172	15.4	(13.4–17.7)	73	13.6	(10.9–16.7)	262	11.4	(10.1–12.7)	571	14.6	(13.5–15.7)
Processed meat	Avoiders	2152	22.4	(21.6–23.2)	357	16.0	(14.6–17.6)	498	56.5	(53.2–59.8)	235	21.1	(18.7–23.5)	163	30.4	(26.6–34.3)	576	25.0	(23.3–26.8)	750	19.1	(17.9–20.4)
	Limiters	4229	44.0	(43.0–45.0)	800	36.0	(34.0–38.0)	383	43.5	(40.2–46.8)	514	46.1	(43.1–49.0)	256	47.7	(43.5–51.9)	1057	45.9	(43.9–48.0)	1809	46.1	(44.5–47.6)
	Includers	3222	33.6	(32.7–34.5)	1068	48.0	(45.9–50.1)	0	0.0	(0.0–0.0)	367	32.9	(30.2–35.7)	118	22.0	(18.6–25.6)	669	29.1	(27.2–30.9)	1367	34.8	(33.3–36.3)
Fish and shellfish	Avoiders	2172	22.5	(21.7–23.3)	532	23.8	(22.0–25.5)	368	42.0	(38.7–45.2)	121	10.8	(9.1–12.8)	109	20.2	(17.0–23.8)	435	18.9	(17.3–20.5)	848	21.5	(20.2–22.8)
	Limiters	4560	47.3	(46.3–48.3)	937	41.8	(39.8–43.9)	380	43.3	(40.1–46.6)	525	47.0	(44.1–50.0)	275	51.0	(46.8–55.2)	1115	48.4	(46.4–50.4)	1950	49.5	(47.9–51.0)
	Includers	2903	30.1	(29.2–31.0)	771	34.4	(32.5–36.4)	129	14.7	(12.5–17.2)	470	42.1	(39.2–45.0)	155	28.8	(25.1–32.7)	754	32.7	(30.8–34.7)	1145	29.0	(27.6–30.5)
Fruit	Avoiders	339	3.5	(3.1–3.9)	103	4.6	(3.8–5.5)	46	5.2	(3.9–6.9)	11	1.0	(0.5–1.7)	27	5.0	(3.4–7.1)	30	1.3	(0.9–1.8)	137	3.5	(2.9–4.1)
	Limiters	1260	13.1	(12.4–13.8)	321	14.3	(12.9–15.8)	191	21.8	(19.1–24.6)	40	3.6	(2.6–4.8)	58	10.8	(8.4–13.6)	114	4.9	(4.1–5.9)	589	14.9	(13.8–16.1)
	Includers	8042	83.4	(82.7–84.1)	1820	81.1	(79.4–82.7)	640	73.0	(70.0–75.8)	1065	95.4	(94.1–96.5)	454	84.2	(81.0–87.1)	2160	93.8	(92.7–94.7)	3219	81.6	(80.4–82.8)
Starchy vegetables	Avoiders	297	3.1	(2.8–3.4)	64	2.9	(2.2–3.6)	61	7.0	(5.4–8.8)	10	0.9	(0.5–1.6)	37	6.9	(5.0–9.2)	27	1.2	(0.8–1.7)	118	3.0	(2.5–3.6)
	Limiters	1315	13.6	(12.9–14.3)	238	10.6	(9.4–11.9)	272	31.1	(28.1–34.2)	66	5.9	(4.6–7.4)	70	13.0	(10.3–16.0)	184	8.0	(6.9–9.1)	595	15.1	(14.0–16.2)
	Includers	8022	83.3	(82.6–84.0)	1940	86.5	(85.1–87.9)	543	62.0	(58.7–65.2)	1040	93.2	(91.6–94.6)	432	80.1	(76.6–83.3)	2093	90.8	(89.6–92.0)	3228	81.9	(80.7–83.1)
Non-starchy vegetables	Avoiders	553	5.8	(5.3–6.3)	145	6.5	(5.5–7.6)	88	10.1	(8.2–12.2)	0	0.0	(0.0–0.0)	0	0.0	(0.0–0.0)	0	0.0	(0.0–0.0)	320	8.2	(7.3–9.1)
	Limiters	1113	11.6	(11.0–12.2)	236	10.6	(9.4–11.9)	210	24.0	(21.3–26.9)	0	0.0	(0.0–0.0)	0	0.0	(0.0–0.0)	0	0.0	(0.0–0.0)	667	17.0	(15.9–18.2)
	Includers	7930	82.6	(81.8–83.4)	1847	82.9	(81.3–84.4)	576	65.9	(62.7–69.0)	1116	100.0	(100.0–100.0)	539	100.0	(100.0–100.0)	2304	100.0	(100.0–100.0)	2932	74.8	(73.4–76.2)
Cakes and biscuits ^4^	Avoiders	584	6.1	(5.6–6.6)	53	2.4	(1.8–3.1)	108	12.3	(10.3–14.6)	59	5.3	(4.1–6.7)	89	16.5	(13.6–19.9)	112	4.9	(4.0–5.8)	254	6.4	(5.7–7.2)
	Limiters	2676	27.8	(26.9–28.7)	256	11.4	(10.2–12.8)	355	40.5	(37.3–43.8)	242	21.7	(19.3–24.2)	224	41.6	(37.5–45.8)	548	23.8	(22.1–25.6)	1367	34.7	(33.2–36.2)
	Includers	6370	66.1	(65.2–67.0)	1932	86.2	(84.7–87.6)	413	47.1	(43.9–50.5)	815	73.0	(70.4–75.6)	225	41.8	(37.7–46.0)	1644	71.4	(69.5–73.2)	2318	58.8	(57.3–60.4)
Nuts	Avoiders	1941	20.2	(19.4–21.0)	434	19.5	(17.9–21.2)	210	23.9	(21.2–26.9)	98	8.8	(7.2–10.5)	123	22.9	(19.5–26.6)	323	14.0	(12.6–15.5)	891	22.6	(21.4–24.0)
	Limiters	3282	34.1	(33.2–35.0)	651	29.2	(27.3–31.1)	294	33.5	(30.5–36.7)	315	28.2	(25.6–30.9)	168	31.2	(27.4–35.2)	739	32.1	(30.2–34.0)	1495	38.0	(36.5–39.5)
	Includers	4393	45.7	(44.7–46.7)	1144	51.3	(49.2–53.4)	373	42.5	(39.3–45.8)	703	63.0	(60.1–65.8)	247	45.9	(41.7–50.1)	1242	53.9	(51.9–55.9)	1550	39.4	(37.9–40.9)
Confectionary: sweets and chocolate	Avoiders	1067	11.1	(10.5–11.7)	48	2.1	(1.6–2.8)	166	19.0	(16.5–21.7)	126	11.3	(9.5–13.3)	123	22.9	(19.5–26.6)	275	11.9	(10.7–13.3)	502	12.8	(11.7–13.8)
	Limiters	3875	40.3	(39.3–41.3)	286	12.8	(11.4–14.2)	444	50.7	(47.4–54.0)	511	45.8	(42.9–48.8)	273	50.8	(46.6–55.1)	1067	46.3	(44.3–48.4)	1925	48.9	(47.4–50.5)
	Includers	4682	48.6	(47.6–49.6)	1909	85.1	(83.6–86.5)	265	30.3	(27.3–33.4)	478	42.9	(40.0–45.8)	141	26.3	(22.7–30.1)	961	41.7	(39.7–43.8)	1507	38.3	(36.8–39.8)
Full sugar soft drinks ^5^	Avoiders	3284	34.1	(33.2–35.0)	293	13.1	(11.7–14.5)	405	46.2	(42.9–49.5)	480	43.0	(40.1–45.9)	302	56.1	(51.9–60.3)	1028	44.6	(42.6–46.7)	1398	35.5	(34.1–37.1)
	Limiters	3038	31.6	(30.7–32.5)	314	14.0	(12.6–15.5)	327	37.3	(34.1–40.5)	436	39.1	(36.2–42.0)	157	29.2	(25.5–33.1)	860	37.3	(35.4–39.3)	1461	37.1	(35.6–38.7)
	Includers	3303	34.3	(33.4–35.2)	1634	72.9	(71.0–74.7)	145	16.5	(14.2–19.1)	200	17.9	(15.8–20.3)	79	14.7	(11.9–17.9)	416	18.1	(16.5–19.7)	1074	27.3	(25.9–28.7)
Takeaways ^6^	Avoiders	1506	15.6	(14.9–16.3)	164	7.3	(6.3–8.4)	246	28.0	(25.1–31.1)	206	18.5	(16.3–20.8)	137	25.5	(21.9–29.3)	437	19.0	(17.4–20.6)	607	15.4	(14.3–16.6)
	Limiters	5542	57.6	(56.6–58.6)	976	43.5	(41.5–45.6)	541	61.6	(58.4–64.8)	736	65.9	(63.1–68.7)	320	59.5	(55.3–63.6)	1517	65.8	(63.9–67.8)	2345	59.6	(58.1–61.1)
	Includers	2581	26.8	(25.9–27.7)	1102	49.2	(47.1–51.2)	91	10.4	(8.5–12.5)	174	15.6	(13.6–17.8)	81	15.1	(12.2–18.3)	350	15.2	(13.8–16.7)	983	25.0	(23.6–26.4)

^1^ Nutrition profiles names based around eating pattern; ^2^ Confidence intervals (CI); ^3^ Avoiders defined as not consuming food groups, Limiters defined as consuming food group a few times a month, Includers defined as consuming food group a few times a week or more often; ^4^ Cakes and biscuits includes, chips, crackers or muesli bars; ^5^ Full sugar soft drinks includes sports drinks, fruit juice or cordial; ^6^ Takeaways includes fast food outlets, and fish and chips; Note, totals do not add to 10,012 as profiles allow for overlap.
